# Accelerating Education During COVID-19 Through Virtual Learning

**DOI:** 10.1093/asjof/ojaa023

**Published:** 2020-05-21

**Authors:** Foad Nahai, Jeffrey M Kenkel

**Affiliations:** 1 Division of Plastic Surgery, Emory University School of Medicine, Atlanta, GA; 2 Department of Plastic Surgery, UT Southwestern Medical Center, Dallas, TX

The curve has begun to flatten and many of us have returned to the office and the operating theater. For many of us, this is likely the longest time in our careers without seeing patients, performing aesthetic procedures, and traveling to meetings. We are glad this is now in our rearview mirror and we can return to work as normal or should we say what we hope will be a temporary new normal?

We are proud of the educational efforts the *Aesthetic Surgery Journal* offered during the lockdown. Through the contributions of many of our lifelong peers and our editorial team, both *ASJ* and *ASJ Open Forum* quickly became a go-to resource during COVID-19.

We must give credit where it is due. Dr. Angela Cheng, Associate Professor of Surgery in the Division of Plastic and Reconstructive Surgery at Emory University School of Medicine, conceived of the idea of “Virtual Grand Rounds” so that residents and fellows would not miss out on learning opportunities during the quarantine. Her brilliant and original idea was to host Zoom meetings 3 times per week, with 2 sessions featuring reconstructive topics and 1 session featuring aesthetic topics. She graciously agreed to allow those of us at *ASJ* to choose the topics and speakers and we called upon our Section Editors. They rose to the challenge and did not disappoint with hour-long talks followed by copious Q&A sessions that in many cases lasted an additional hour. Dr. Cheng moderated all of these sessions with her own unique style and acumen, curating the questions with assistance from our colleague Dr. William J. Knaus (Assistant Professor of Surgery, Emory University School of Medicine). She even scrubbed out during 1 session to moderate, which is testimony to her passion and commitment to educating the next generations. In some form or fashion, the educational opportunity conceived by Dr. Cheng will continue after COVID and we at *ASJ* intend to explore options for furthering her efforts in the future.

On the heels of the success of *ASJ* Virtual Grand Rounds, our Executive Editor Phaedra Cress conceived of *ASJ* GEMS (Global Educational Meetings)—Zoom meetings featuring experts and targeting the international community. As international submissions continue to soar, we know there is so much we can teach and learn from the global community. *ASJ* GEMS tackled topics ranging from eyelid complications to managing vaginal rejuvenation patients, plastic surgery practices after COVID-19, and body contouring after massive weight loss. Engaging with our international aesthetic colleagues from all 7 continents brought the presentations and discussions to a new and also gave us the opportunity to promote our new open-access journal, *ASJ Open Forum*, which is free to read for everyone. Allergan graciously agreed to support several of the *ASJ* Virtual Grand Rounds sessions, and we would like to offer our sincere thanks for promoting *ASJ*’s educational efforts during this challenging time.

Many of the *ASJ* Virtual Grand Rounds and *ASJ* GEMS sessions were recorded and are available on Radar Resource for journal subscribers and The Aesthetic Society members. We know these videos will provide valuable knowledge for many years to come.

The *ASJ* Journal Club also continues to thrive with record attendance at the May session that featured authors such as Dr. Frank Lista, *ASJ* Breast Section Co-Editor, Dr. Bradley Calobrace, and our new lead Next Generation Editor, Dr. Ryan Austin. These sessions are available on the *ASJ* website and our YouTube channel. They will continue to be held on the first Tuesday of each month at 8:00 pm EDT (Eastern Daylight Time). 

Finally, we salute The Aesthetic Society who convened a task force chaired by Dr. James Fernau making formal recommendations on reopening the office and resuming elective procedures. They have also held weekly webinars on COVID-19 and have made those recordings available free to all. The task force recommendations and webinars are available here: https://www.surgery.org/private/webinars.

In addition to these virtual learning resources, we thank Dr. Tom Fiala who served as Guest Editor for a thematic issue entitled: “Practice Management and Getting Back to Work After a Pandemic” that can be read here https://academic.oup.com/asj/pages/practice-management-thematic-issue and includes practice management articles by leading aesthetic surgeons, such as Dr. Jeffrey Kenkel, Dr. Guy Massry, Dr. James Zins, Dr. Jamil Ahmad, Dr. Greg Evans, and many others.

Thank you for continuing to support *ASJ* and *ASJ Open Forum* and we wish you all continued safety and good health. We know we will meet again “face-to-face” at an upcoming conference and we welcome that opportunity.

## Disclosures

This article has been co-published with permission in the *Aesthetic Surgery Journal* and *Aesthetic Surgery Journal Open Forum*. The articles are identical and either citation can be used when citing this article.

## Funding

The authors received no financial support for the research, authorship, and publication of this article.

